# Specific-mutational patterns of p53 gene in bladder transitional cell carcinoma among a group of Iraqi patients exposed to war environmental hazards

**DOI:** 10.1186/1756-0500-5-466

**Published:** 2012-08-28

**Authors:** Thekra A Al-Kashwan, Massoud Houshmand, Asaad Al-Janabi, Alice K Melconian, Dhafir Al-Abbasi, Muhammad N Al-Musawi, Maryam Rostami, Akeel A Yasseen

**Affiliations:** 1 Middle Euphrates unit for cancer research, Faculty of Medicine, University of Kufa, Kufa, Iraq; 2 National Institute for Genetic Engineering and Biotechnology, Tehran, Iran; 3 Department of Pathology and Forensic Medicine, Faculty of Medicine, University of Kufa, Kufa, Iraq; 4 Biotechnology Department, College of Science, Baghdad university, Baghdad, Iraq; 5 Urology department, Faculty of Medicine, University of Kufa, Kufa, Iraq; 6 Department of Pathology and Forensic Medicine, Faculty of Medicine, University of Kufa, Kufa,, P.O. Box 21, Najaf Governorate, Iraq

**Keywords:** Bladder cancer, TP53 alteration, Specific mutation, Immunohistochemistry

## Abstract

**Background:**

To unfold specific-mutational patterns in TP53 gene due to exposures to war environmental hazards and to detect the association of TP53 gene alteration with the depth of bladder cancer.

**Methods:**

Twenty-nine bladder carcinomas were analyzed for TP53 alterations. PCR-single strand conformational polymorphism analysis, DNA sequencing and immunohistochemical analysis using monoclonal mouse anti-human *p53* antibody (Clone DO-7) were employed.

**Results:**

TP53 gene mutations occurred in 37.9% of the cases while TP53 overexpression occurred in 58.6%. Both of them were associated with deep invasive-tumors. Single mutations were seen in 63.6%, whereas only 27.3% have shown double mutations. Four mutations were frameshifted (30.8%); two of them showed insertion A after codon 244. There was no significant association between TP53 mutations and protein overexpression (P>0.05), while a significant association was observed between TP53 alterations and tumors progression (P ≤ 0.01).

**Conclusion:**

The infrequent TP53mutations, especially insertion A and 196 hotspot codon, may represent the specific-mutational patterns in bladder carcinoma among the Iraqi patients who were exposed to war environmental hazards. TP53 alteration associated with bladder cancer progression should be analyzed by both mutational and protein expression analysis.

## Background

Bladder cancer (BC) is the most common malignancy affecting urinary system comprising the seventh most common cancer worldwide with male predominance [1,2]. In Iraq, the incidence of bladder cancer is the fourth most common type of cancer (in men and eighth most common in women) [3]. The most common type of bladder cancer is transitional cell carcinoma (TCC) accounting for more than 90% of the cases [2]. It represents one of the first tumors that have been associated with environmental risk factors that produce genetic alterations [4,5].

TP53 gene “the guardian of genome” is the most frequently mutated tumor suppressor gene identified in human cancer. TP53 inactivation led to diminished control cell cycle check points, decreased DNA repair, and increased genomic instability [6-8] . Furthermore, TP53 inactivation has been identified in tumor progression, metastasis, and aggressive phenotype of bladder cancer giving rise to what has been considered as a useful genetic biomarker to predict progression associated with bad prognosis [9-11]. The frequency and the type of mutations vary from one tumor type to another, ranging from 5% to 80% depending on the type, stage and etiology of tumor [12]. Almost all TP53 mutations are point missense mutations leading to a functionally defective protein [13]. Approximately 90% of them are localized in DNA-binding domains encoded by exons 5–8. In total, about 40% of them are localized at the "hotspot" residues R 175, G 245, R 248, R 249, R 273 and R 282 [14]. Indeed, there are considerable numbers of studies on TP53 gene being as a target for carcinogens, with a specific TP53 mutations spectrum, mainly G: C→A: T transitions at CpG and non-CpG sites in bladder cancer [7,15,16]. It is well documented that the spectrum of TP53mutations in bladder cancer differs from that of lung cancer, even though cigarette smoking is probably a contributing cause in over one-third of all bladder cancer cases [17]. Specifically, G→T transversions at CpG are relatively uncommon (about 8% in bladder cancer versus 27% in lung cancer) whereas CpG G: C→A: T transition are as twice as common with regard to mutation patterns (22% for bladder cancer versus 11% for lung cancer) [7].

Inactivation of tumor suppresser genes can occur either primarily through mutations, or without any change in the structure of the given genes. Thus, tumor suppressor function should be analyzed at the level of the genes as well as at the level of proteins, and in the context of the pathways in which these genes are involved [18,19]. The mutated p53 protein has a longer half-life, as compared with normal *p53* protein, which can be detected by Immunohistochemistry (IHC) as a surrogate marker for mutation [20]. Immunohistochemical positivity for TP53protein is in general thought to reflect point mutations of TP53 gene in tumor, although it is not always synonymous with TP53 mutation [21]. Accordingly, using both molecular and protein analyses (PCR-based genetic technique and immunohistochemistry respectively) for detection of TP53 alterations have rational efficacy for mutation detection rather than each one alone [20,22,23]. The aims of this research were to determine if any specific-mutational pattern in TP53gene may play a possible role in bladder tumorogenesis, which might be resulted from the exposure to the hazardous pollution of the wars in Iraq. An analysis of the alteration of TP53gene using combined PCR-based genetic and IHC analysis to evaluate its association with bladder cancer progression was conducted.

## Methods

Ethical approval was obtained from the local medical ethics committee, Faculty of Medicine, University of Kufa. A written informed consent was received from all subjects before proceeding any further. The study was designed and conducted in accordance with the tents of Declaration of Helsinki.

A total of twenty-nine patients (25 males and 4 females) with transitional cell carcinoma (TCC), diagnosed by transurethral resection (TUR-biopsy) at the Department of Pathology of Kufa School of Medicine Teaching Hospital were subjected to the present study. The patients were randomly selected from the Middle Euphrates area and south of Iraq. Both regions were potentially exposed to environmental pollution during the last two decades of wars. The patients ages ranged between 35 and 85 years, with a median age of 69.3 years. Histological examination, grading [24] and staging [25] were performed by two of us independently. Three were classified as grade I, two as grade II, and fifteen classified as grade III. Fourteen cases were superficial TCC [5 as Ta and 9 as T1], while 15 cases were deep invasive TCC (T2). Sixteen of our patients had a history of cigarette smoking for at least 10 years.

### TP53 status

#### TP53 protein expression

TP53 protein expression was estimated using immunohistochemical techniques [26-28]. Five micron thick sections of formalin-fixed paraffin-embedded tissue (FFPE) were placed on positively charged slides (Fisher scientific Co., Pittsburgh, PA). These sections were then deparaffinized and rehydrated. For staining enhancement, the sections were pre-treated with antigen retrieval solution (0.01 M, citrate buffer, pH6.0, Dako Cytomation/Denmark) in water-bath at 95°C for 40 minutes followed by staining with a monoclonal mouse anti-human TP53 antibody (Clone DO-7, Ready-to-use, DakoCytomation/Denmark). The antigen-antibody complex was visualized using Labeled Streptavidin-Biotin 2 System-Horseradish Peroxidase staining technique (LAB/LSAB2 System-HRP, DakoCytomation/Denmark). The sections were then counterstained with Meyer's haematoxylin.

A breast cancer with high TP53expression detected by immunohistochemical analysis was used as positive external control. Negative controls were obtained by omission of the primary antibody and by a breast cancer with negative TP53 expression detected by immunohistochemical analysis. The non-epithelial cells of samples (lymphocytes, stromal cells and endothelial cells) were used as negative internal control.

All slides were reviewed independently by two investigators without previous knowledge of tumor grade and stage or TP53mutation. TP53 expression (nuclear staining) was evaluated by counting 100 cells/section in five randomly chosen high-power fields (40x) by light microscope. The extent of nuclear reactivity was classified in four categories [9,20]; no nuclear reactivity (−), few focally positive cells (1 to 10% tumor cells) (+/−), heterogeneous nuclear reactivity (10 to 50% tumor cells) (+) and homogenous intense nuclear reactivity (50 to 100% tumor cells) (++). The samples which demonstrated at least 10% nuclear reactivity were considered to be TP53-positive (have an alteration in TP53) [9,10,19,20].

### TP53 mutation

#### DNA extraction

High-molecular weight DNA was prepared from the fresh tumor specimens by phenol-chloroform-isoamyl alcohol method [20,29]. DNA which was extracted from whole blood samples of healthy looking individuals was included as normal control with their matched age and sex with Wizard ® Genomic DNA purification kit (Promega Company/USA).

### PCR-SSCP and DNA sequencing

The mutational analysis was achieved using PCR-single strand conformation polymorphism (PCR-SSCP) and direct DNA sequencing methods for TP*53* gene exons 5–8. Each exon 5–8 was amplified by PCR. The primer sequences used were as follows [15]: (1) exon 5/228 bp, forward: 5′-TTCAACTCTGTCTCCTTCCT-3′ and reverse: 5′–CAGCCCTGTCGTCTCTCCAG-3′; (2) exon 6/159 bp, forward: 5′– GCCTCTGATTCCTCATCGAT-3′ and reverse:5′–TTAACCCCTCCTCCCAGAGA-3′; (3) exon 7/157 bp, forward:5′-AGGCGCACTGGCCTCATCTT-3′ and reverse:5′-TGTGCAGGG TGG CAAGTGGC-3′; and (4) exon 8/214 bp, forward:5′– TTCCTTACTGCCTCTTGCTT-3′and reverse: 5′–AGGCATAACTGCAC CCTTGG-3′. Each PCR reaction was performed in a final volume of 25 μl containing 100 ng DNA, 1X PCR buffer, 1.5 mM MgCL2, 200 μM each dNTP, 0.1 μM of each upstream and downstream primer, and 1.5u of Taq polymerase (CinnaGen Company/Iran). PCR was carried out under the following conditions: an initial denaturation step (95°C for 5 minutes) was followed by 35 cycles consisting of (for exon 5) denaturation at 95°C for 50 seconds, primer annealing at 55°C for 35 seconds and extension at 72°C for 30 seconds; (for exons 6–7) denaturation at 95°C for 50 seconds, primer annealing at 63°C for 25 seconds and extension at 72°C for 15 seconds; or was followed by 30 cycles (for exons 8) consisting of denaturation at 95°C for 50 seconds, primer annealing at 65°C for 25 seconds and extension at 72°C for 15 seconds. The final extension was continued for 10 minutes at 72°C. The PCR products were analyzed on 1.5-2% agarose gel to determine the specific band of each exon product and then analyzed on 12% polyacrylamide gel to another evaluation of specific band purity to ensure the absence of any unwanted products (non-specific bands) which may interfere with SSCP and DNA sequencing analysis.

For non-radioactive SSCP analysis, the SSCP analysis was carried out according to the method of Liechti-Gallati *et al.*, (1999) [30], for both of the tumor and the control samples.

The to controls were analyzed. PCR products were denaturated at 96°C for 10 min at 3:2 dilution of formamide loading dye (SSCP denaturing solution) containing 95% formamide, 100 mM NaOH, 0.25% bromophenol blue, 0.25% xylene cyanol and thereafter placed immediately on ice to prevent re-annealing of the single-stranded product. The denaturized samples, the controls and the normal PCR product of controls were loaded quickly into wells of the 12% polyacrylamide gel and run at 4–10°C/80 V for overnight. The gel was stained with Sliver Staining and alterations of bands relative

All the samples that revealed mobility shift in their migration during SSCP screening mutation analysis were sent to the direct DNA sequencing in both directions: forward sequencing 5′→3′and reverse sequencing 3′→5′using the same primer sequences and dideoxy chain termination method. The DNA sequencing was achieved by Gen Fanavaran Company/Iran. The DNA sequencing chromatogram was interpreted using Chromagen 2.3 version software that allows comparison of a newly generated sequence of DNA sample with free DNA sequence. Viewers (available from National Center for Biotechnology Information (NCBI) website) were used as reference sequence of gene of interest for comparison.

### Statistical analysis

The Fisher’s exact probability test and Odds ratios (ORs), using contingency tables, were applied to analyze the data using the program statistical package for the Social Science (SPSS for windows, version 10.0). The relationships between the variables were assessed using non-parametric Fisher’s exact probability test. A P-value ≤0.05 was considered as statistically significant at a level of 5%. The strength of the associations between the variables was measured by calculating Odds ratios (ORs) and confidence intervals (95% CI). Possible categories for OR are greater than 1and less than 1. A value greater than 1 indicates positive association and a value less than 1 indicates negative association.

## Results

### Mutational analysis of the TP53 gene in relation to clinicopathological features

#### Characteristic of TP53 gene mutation

**Figure 1:**
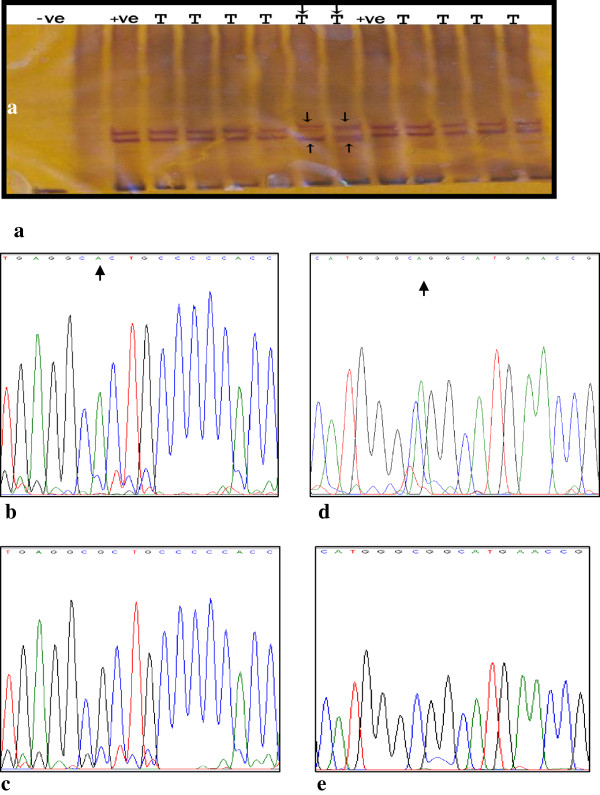
**Example of PCR-SSCP/HD and DNA sequencing analysis of p53 gene in transitional cell carcinoma.** (**a**) PCR-SSCP/HD analysis of exon 5 shows extra bands and bands with mobility shift indicated by arrows (−ve: undenaturated DNA control; +ve: denaturated DNA control, and T: tumors). DNA sequencing reveals transition CGC → CAC at codon 175 in exon 5 (**b**) and insertion A after codon 244 (GGC) in exon in exon 7 (**d**) in compared to wild types (**c**) and (**e**) respectively (arrows).

The PCR-SSCP analysis showed that 12 out of 29 cases of bladder cancer patients had aberrantly migrating bands or extra bands that were further analyzed by DNA sequencing to represent the mutations of TP53 gene (Figure 1). All controls used in this study revealed normal migration of SSCP bands and normal sequencing of all studied exons (Figure 1a,c,e).

The sequencing results confirmed that 10 cases harbored one or more TP53 mutations within identical or separated samples, one case had silent mutation and one case had bad sequencing. Eleven (37.9%) cases were classified as TP53-positive [10 cases with detected mutation and 1 case without DNA sequencing] and 18 (62.1%) cases as *p53*-negative [17 cases without detected mutation and 1 case with silent mutation: case 5, codon 244; GGC (Gly) to GGA (Gly)] (Table 1).

**Table 1:** Mutational analysis of the p53 detection by SSCP and DNA sequencing, p53 nuclear reactivity and p53 status

**Patients No**	**Exon/Codon**	**Codon change**	**Base change**	**Mutation type**	**Amino acid change**	**p53 nuclear reactivity**	**p53 status**
1	-	-	-	-	-	-	-
2	-	-	-		-	-	-
3	5/184	GAT→AAT	G→A	Ts	Asp184Asn	++	+
4	-	-	-		-	-	-
5	7/244	GGC→GGA	C→A	Tv	Gly244Gly	-	-
6	7/230	Del A	-	Fr	-	-	+
7	7/NS	-	-		-	++	+
8	-	-	-		-	-	-
9	-	-	-		-	++	+
10	-	-	-		-	++	+
11	-	-	-		-	-	-
12*	5/154	GGC→GGA	C→A	Tv	Gly154Gly	+	+
	6/192	CAG→CAC	G→C	Tv	Glu192His		
13	7/244	+ A	-	Fr	-	-	+
14	-	-	-	-	-	-	-
15	7/244	+ A	-	Fr	-	-	+
16	-	-	-	-	-	++	+
17	-	-	-	-	-	+	+
18	-	-	-	-	-	+	+
19	-	-	-	-	-	++	+
20*	6/196	CGA→CAA	G→A	Ts	Arg196Glu	++	+
	8/283	CGC→CCC	G→C	Tv	Arg283Pro		
21	5/175	CGC→CAC	G→A	Ts	Arg175His	++	+
22	5/176	TGC→GGC	T→G	Tv	Cys176Gly	++	+
23	-	-	-	-	-	++	+
24	-	-	-	-	-	-	-
25*	8/293	Del G	-	Fr	-	-	+
	8/294	GAG→GAA	G→A	Ts	Glu294Glu		
26	6/196	CGA→CAA	G→A	Ts	Arg196Glu	++	+
27	-	-	-	-	-	++	+
28	-					++	+
29	-	-	-		-	++	+

p53 status; + has alteration (mutation) or immunoreactivty +,++ (IHC), -; no alteration (mutation (−) and IHC (−,+/−), Ts; Transition, Tv; Transvertion, *; Tumor with double mutation, NS; DNA not sequenced but p53 mutation detected by SSCP analysis, - G; deletion of one base (G) at codon 293, - A; deletion of one base (A) at codon 230, + A; Insertion of one base (A) after codon 244/exon 7, Fr; Frameshift.

Among TP53-positive cases; seven patients (63.6%) showed single mutation, three patients (27.3%) had double mutations and one patient (9.1%) with mutation detected only by SSCP analysis. A total of 13 mutations determined in ten cases; 9 mutations (69.2%) were single-base pair substitutions and 4 mutations (30.8%) were with frameshift mutations.

The patterns of TP53 base-pair mutations showed that five mutations (38.4%) were of transitions including G: C → A: T, three of them (23.8%) occurred at CpG dinucleotide in the codons (175 and 196) that were reported as hotspot for TP53 mutations. The other two (15.4%) mutations occurred at non-CpG sites. The four (30.8%) transversions detected were two (15.4%) G → C, one (7.7%) T → G, and one (7.7%) C → A. Two of the base-pair substitutions were silent mutations found in double mutations [case 12, codon 154; GGC (Gly) to GGA (Gly) and case 25, codon 294; GAG (Glu) to GAA (Glu)], and seven were missense resulted in amino acid changes.

Of the observed frameshift mutations (30.8%) three (23.1%) were in the exon7 [two (15.4%) with insertion A after the codon 244 (GGC/Gly) and one (7.7%) with deletion A at the codon 230(ACC/Thr)]. The remaining one was deletion G (7.7%) at codon 293 (GGG/Gly) in exon 8. The double mutations were found in case 12 in exon 5 [codon 154; Gly → Gly (silent)] and in exon 6 [codon 192; Glu → His (missense)], case 20 in exon 6 [codon 196; Arg → Glu (missense)] and exon 8 [codon 283; Arg → pro (missense)], and case 25 in exon 8 [codon 293, del G and codon 294, Glu → Glu (silent)] (Table 1) (Figure 1b and d). The TP53 mutations were higher in deep invasive-tumors (high grade and stage T2) (40%) than in superficially invasive tumors (low grade and stage Ta-T1) (35.7%) (OR, 1.2; CI, 0.26-5.4) (Table 2).

**Table 2:** Association between p53 mutations as analyzed by SSCP and DNA sequencing, p53 immunoreactivty and p53 status with clinicopathological features

**Variable**	**Patients No.**	**p53 mutation (5-8 exons) No. (%)**				**p53 nuclear reactivity No. (%)**							**p53 status No. (%)**		
		**p53 mut**	**p53 wt**	**OR**	**95%CI**	**Negative**			**Positive**			**P value**	**altered**	**Non- altered**	**P value**
						**-**	**+/-**	**Total**	**+**	**++**	**Total**				
Total	29	11(37.9)	18(62.1)			6(20.7)	6(20.7)	12(41.4)	3(10.3)	14(48.3)	17(58.6)		21(72.4)	8(27.6)	
Grade															
Low(I&II)	14	5(35.7)	9(64.3)	1.2	0.26-5.4	4(28.6)	5(35.7)	9(64.3)	1(7.1)	4(28.6)	5(35.7)	0.02	7(50)	7(50)	0.01
High (III)	15	6(40)	9(60)			2(13.3)	1(6.7)	3(20)	2(13.3)	10(66.7)	12(80)		14(93.3)	1(6.9)	
Stage															
Low(Ta&T1)	14	5(35.7)	9(64.3)	1.2	0.26-5.4	4(28.6)	5(35.7)	9(64.3)	1(7.1)	4(28.6)	5(35.7)	0.02	7(50)	7(50)	0.01
High (T2)	15	6(40)	9(60)			2(13.3)	1(6.7)	3(20)	2(13.3)	10(66.7)	12(80)		14(93.3)	1(6.9)	

Negative indicates that less than 10% of tumor nuclei demonstrated p53 immunoreactivty, positive; 10% or more of tumor nuclei demonstrated p53 immunoreactivty, p53 status; + has alteration (mutation, or immunoreactivty score +,++, or both), - no alteration (mutation (−) and IHC (score -, +/−) for p53), *OR*, Odds Ratio; *CI*, Confidence Intervals, P Value ≤ 0.05 or 0.01 (Fisher’s exact probability test).

#### Immunhistochemical analysis of TP53 gene in relation to clinicopathological features

**Figure 2:**
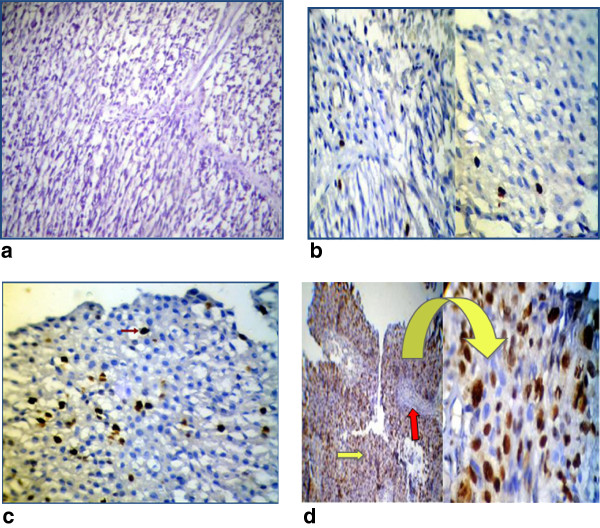
**Immunohistochemical detection of p53 nuclear reactivity in transitional cell carcinoma (a) Invasive transitional cell carcinoma, poorly differentiated (Grade III) showing no detectible nuclear p53 immunostaining (Score (−), (10X)).(b)** Papillary transitional cell carcinoma, well differentiated (Grade I) showing a few p53 immunoreactivty of tumor nuclei (Score (+/−), arrowed, (10X and 40X)). **(c)** Invasive transitional cell carcinoma, poorly differentiated (Grade II) showing heterogeneous p53 nuclear immunostaining (Score (+); arrowed,(40X)). **(d)** Papillary transitional cell carcinoma, well differentiated (Grade I) showing homogenous intense p53 nuclear immunostaining (Score (++); yellow arrow, (10X and 40X), (Red arrow indicates surrounding stromal and infiltrative lymphocytes with no detectible nuclear p53 immunostaining)).

Immunohistochemical analysis showed that TP53 immunoreactivty was restricted to the nuclei of tumor cells. Four patterns of immunohistochemical nuclear staining was observed (Figure 2) including: no detectable immunoreactivty (−) in 20.7% (Figure 2,a), few focal reactivity in less than 10% of tumor cells (+/−) in 20.7% (Figure 2,b), heterogeneous nuclear reactivity in 10 -50% of tumor cells (+) in 10.3% (Figure 2,c), and intense homogenous nuclear reactivity was greater than 50% of tumor cells (++) in 48.3% (Figure 2,d). The level of TP53 nuclear reactivity was classified into two categories: wild-type TP53including score (−) and (+/−), and altered TP53 includes score (+) and (++). While, negative internal control of samples (non-epithelial cells) showed absence of TP53 nuclear staining that was consistent with wild-type expression of TP53 gene (Figure 2, D). The TP53 overexpression was identified in 17 cases (58.6%) out of 29 TCC cases. The altered expression of TP53 was more frequent in high grade tumor than low grade (66.7% vs. 28.6% respectively) and in T2 stage than Ta and T1 (66.7% vs. 28.6% respectively), giving rise to a statistically significant difference (p *≤* 0.05) (Table 2).

#### Correlation of p53 mutation with nuclear reactivity

The present data shows no significant association between TP53 mutations and the immunohistochemical detection of TP53 protein (p>0.05) (Table 3). Seven tumors which had TP53 mutation [six of them with point mutation (missense), and one case with SSCP mobility shift] were positive for TP53protein overexpression. Four tumor cases with frameshift mutations of TP53had no detectable TP53 nuclear accumulation. Ten tumor cases without detected mutation had TP53overexpression. Accordingly, the TP53 alteration (TP53 status) defined by either presence of mutations or TP53 immunoreactivty, or both was demonstrated (Table 1) [23]. Our data showed that the TP53 alteration (TP53 status) was strongly associated with high grade and stage (p≤ 0.01) (Table 2).

**Table 3:** Relation of p53 immunoreactivty to p53 mutation analyzed by SSCP and DNA sequencing

	**Total**	**p53 nuclear reactivity No. (%)**		**P value**
		**Positive**	**Negative**	
p53 mutation No. (%)	29	17(58.6)	12(41.4)	
p53 mut	11(37.9)	7(63.7)	4(36.3)	0.09
p53 wt	18(62.1)	10(55.6)	8(44.4)	

P-value> 0.05 (Fisher’s exact probability test); *Mut*, mutant; *wt*, wild-type.

## Discussion

In Iraq, the incidence of most types of cancer (including bladder cancer) has increased sharply in the last few years due to exposure to wars pollution [31-33]. Mutational analysis of the TP53 gene provides a unique opportunity to investigate the etiology, epidemiology, and pathogenesis of human cancer [34,35]. Few studies have been conducted on the Iraqi population following the conflicts that led to hazardous environmental pollution. The effects of exposure to such pollutants is hard to ascertain as it is not possible to compare to data pre-conflict, which are not available either because they were not measured or, because they were destroyed. There is also the difficulty in finding a cohort of unexposed individuals: the conflicts were country wide and the shifting sands spread chemical pollutants over great distances. A definitive geographical location of where the greatest chemical pollution exists could not be determined [31,32]. However, it is not implausible to suggest that almost all Iraqis have had some exposure to hazardous and/or toxic substances. To the best of our knowledge, the present study is the first molecular analysis of TCC in Iraq to determine the pattern of TP53 mutations. The results were compared with other areas polluted with radioactive substances [15,35] and with reported specific TP53 mutations that were known to be associated with smoking, as smoking is a major risk factors for bladder cancer [5].

The present investigation revealed that the frequency of TP53 mutations was higher in deep invasive-tumors (high grade and stage T2) than superficially invasive tumors (low grade and stage Ta-T1) (37.9% Vs 35.7%) (OR, 1.2; CI, 0.26-5.4) (Table 2). These observations provide further support for the proposed association between TP53 alteration and bladder cancer progression [5,20,36]. In these results, a history of smoking was not associated with a high frequency of TP53 mutation, nor with the pattern of mutation in patients with TCC.

Although the number of our patients is rather small, many interesting observations are apparent. Our results showed that the TP53 double mutations constituted 27.3% of all mutations detected, which is consistent with values reported by Yamamoto *et al.*, (1999) [15] who found double mutations in their cases of dysplasia and *CIS* in radio-contaminated areas. The possible explanation for the occurrence of double mutations found in our study is most likely because of the presence of a strong carcinogenic insult from war pollution that may have resulted in multiple transformation events [15,37].

It has been indicated that G → A transitions were the most prevalent type of TP53 mutation in bladder cancer; about half of these transitions occurred at CpG sites [38]. There is increasing evidence to indicate that CpG bases might be more susceptible than other sites to attack by environmental mutagens [39]. Studies conducted in radio-contaminated regions in Ukraine found a high frequency of CpG/G → A mutations (73%) that were significantly different from those reported by IARC [17]. In the present study, the frequency of all G → A transitions was 38.4% and those that occurred at CpG sites were 23.8% with predominant hotspots (2 out of 3 cases) at codon 196. This is considered to be infrequent in bladder tumors [40] and may reflect the effect of certain exogenous carcinogens, such as depleted uranium, on the frequency of mutation (Table 1).

Conversely, the transversion type of mutation that was shown in this study (30.8%) was similar to a previously reported finding which confirmed that TP53 mutation in bladder cancer patients who smoked consisted of G:C → C:G transversions [41,42]. In our study, most TP53 mutations occurred in both smokers and non-smokers (such as G → C transversions), or occurred in non-smokers only (such as C → A and T → G transversions). This observation unveils new evidence for the presence of risk factors for cancer, other than cigarette smoking, that may underlie this type of mutation.

Another important observation was the frameshift mutations which were found to be higher (30.8%) in frequency than what has been reported previously [5,43], and was found preferentially to occur in exon-7 . Two the frameshift mutation having the same mutation (insertion A after codon 244) that cannot be excluded as a relative hotspot (Table 1). In fact, the most common mutations observed in TP53 DNA binding (exons 5–8) were missense mutations while frameshift types were found to be less frequent [43]. Most studies concerning the mutational signature of *p53* in relation to the history of smoking revealed that the frameshift mutation in the DNA binding either have not been observed [44] or occurred less frequently than point mutation (7% and 7.1%) with different types of deletions and insertions [7,40]. In radio-contaminated regions in Ukraine, all TP53 mutations determined were single-bp substitutions; no base deletions or insertions were found [15,45]. This indicated a possible distinct molecular carcinogenesis pathway for bladder cancer after the Chernobyl disaster, based on a different incidence of p53 gene mutations compared with tumors found in the same population before the accident [45].

Accordingly, frameshift mutations (especially insertion A) of TP53 may reflect the effect of certain exogenous environmental contamination and may be considered as a useful predictor marker for bladder cancer. No reports on the levels of environmental pollution from toxic chemicals or radiation exist as no studies have been conducted to measure and specify these effects with accuracy. This study and future studies on the health of the Iraqi populace are needed to stimulate investigations on the extent and localization of environmental pollution caused by the recent conflicts.

In fact, the occurrence of TP53mutations leads to conformational changes of the protein, resulting in a prolonged half-life and subsequent accumulation of mutations in the nuclei. The extended half-life of the protein is the basis for immunohistochemical detection of TP53 [20,22,44]. Whilst immunohistochemical positivity for TP53 protein generally reflects point mutations of TP53 genes in tumor cells, it is not always synonymous with mutations [20,22]. The present study revealed that the TP53 overexpression (58.6%) was observed more frequently in high grade and in T2 stage than in low grade and Ta and T1 tumors (p *≤* 0.05) (Table 2). This is consistent with other findings which reported that the TP53 overexpression was associated with high grade and stage of bladder cancer [20,46].

It has been reported that there is a good concordance between overexpression and mutation of TP53 gene [20,22], however, other studies have shown a considerable discrepancy between them [47,48]. Analysis of the data in this study showed no significant association between TP53 mutations and the immunohistochemical detection of TP53 protein (p>0.05) (Table 3). This is largely because of the four tumors with frameshift mutations that had no detectable TP53 protein. These mutations encoded deleted or truncated proteins that are very unstable in the cell and usually not detectable by IHC method even when using an antibody containing the corresponding N-terminal epitope [49,50]. The tumors with missense mutations were positive for TP53 protein overexpression (Table 3); missense mutations resulting in amino acid change render the TP53 protein a more stable compound with a longer half-life that can be detected by standard immunhistochemical methods [20,44].

On the other hand, the presence of other tumors that demonstrated TP53 overexpression without mutations might be due to mutations in the TP53 that occurred outside exons 5–8 [49], or to the overexpression of TP53 caused not only by TP53 mutations but also by other factors (such as *MDM2*) which bind to TP53 protein, thus increasing its half-life and allowing it to accumulate in the nucleus. This observation may reflect alterations in the TP53 pathway rather than in the TP53 gene itself [6,51]. However, the antibody used in our study recognises both the wild-type and the mutant forms of TP53 protein at the same time. Hence, it is possible that the IHC assay detects accumulation of the wild-type in some cases. The level of wild-type TP53 protein can be increased in response to DNA damage, hypoxia, oncogene activation and changes in the nucleotide pool, which are commonly observed in primary tumors [51,52]. Furthermore, up-regulation of wild-type TP53 protein in tumors may indicate the last step of defence before metastasis [53]. Therefore, the TP53 alteration (TP53status) that is used as a prognostic marker of bladder tumorigenesis should be determined by combination of mutational and IHC analysis. Accordingly, the present investigation of TP53 status has shown a strong association with deep invasive tumors (p≤ 0.01) (Table 2). The altered expression of TP53 tumor suppressor gene is an independent predictor of bladder cancer progression when examined as an individual determinant [10,20].

## Conclusions

The TP53 frameshift mutations (especially insertion A) and 196 hotspot codon may represent a possible specific-mutational patterns associated with bladder tumorigenesis, and reflect a preferential target for exogenous carcinogens. Furthermore, the difference in incidence and type of TP53 mutations among the Iraqi TCC patients may explicitly indicate a distinct molecular pathway responsible for the development of bladder cancer due to exposure to environmental hazards (e.g. depleted uranium). The unusual mutation patterns of TP53 necessitates a complete molecular epidemiological study for further clarification of distinct molecular pathways for bladder cancer pathogenesis among Iraqi patients. The current study confirmed that the combination of molecular analysis and protein expression of TP53 tumor suppressor gene is highly recommended for studying gene alterations in bladder cancer rather than the application of a single approach.

## Abbreviations

LSAB+, Labeled Streptavidin-biotin; BC, Bladder Cancer; TCC, Transitional Cell Carcinoma; FFPE, Formalin-Fixed Paraffin-Embedded Tissue; DU, Depleted Uranium; IHC, Immunohistochemistry; PCR, Polymerase chain reaction; TUR, Transurethral; SSCP, Single strand conformation polymorphism.

## Competing interests

The authors declare that they have no competing interests.

## Authors’ contributions

ThAA and MH carried out the molecular genetic studies. THAA performed the statistical analysis. AA and AKM initiated the project at the University of Kufa . DA and AA carried out the immunohistochemical analysis and histopathological examination. MR participated in the molecular genetic studies. MNA provided the surgical specimens. AAY and AA conceived of the study, participated in its design and drafted the manuscript. All authors read and approved the final manuscript.
